# Data on SEM, TEM and Raman Spectra of doped, and wool carbon nanotubes made directly from CO_2_ by molten electrolysis

**DOI:** 10.1016/j.dib.2017.08.013

**Published:** 2017-08-17

**Authors:** M. Johnson, J. Ren, M. Lefler, G. Licht, J. Vicini, S. Licht

**Affiliations:** Dept. of Chemistry, George Washington University, Washington, D.C. 20052, United States

## Abstract

This SEM, TEM and Raman Spectra and economic calculations data provides a benchmark for carbon nanotubes synthesized via molten electrolyte via the carbon dioxide to carbon nanotube (C2CNT) process useful for comparison to other data on longer length C2CNT wools; specifically: (I) C2CNT electrosynthesis with bare (uncoated) cathodes and without pre-electrolysis low current activation. (II) C2CNT Intermediate length CNTs with intermediate integrated electrolysis charge transfer. (III) C2CNT Admixing of sulfur, nitrogen and phosphorous (in addition to boron) to carbon nanotubes, and (IV) Scalability of the C2CNT process. This data presented in this article are related to the research article entitled “Carbon Nanotube Wools Made Directly from CO_2_ by Molten Electrolysis: Value Driven Pathways to Carbon Dioxide Greenhouse Gas Mitigation” (Johnson et al., 2017) [1].

**Specifications Table**

**Table:** 

Subject area	*Chemistry, Climate Change Mitigation*
More specific subject area	*Carbon Nanotubes and Climate Change Mitigation*
Type of data	*Tables, Figure, Text File*
How data was acquired	*PHENOM Pro-X SEM with EDS or FEI Teneo LV SEM, JEM 2100 LaB6 TEM, LabRAM HR800 HORIBA Raman Spectrometer*
Data format	*Raw and analyzed data is presented*
Experimental factors	*Analyzed cathode product is washed to remove congealed electrolyte*
Experimental features	*CO*_*2*_*is electrolyzed in molten carbonate forming carbon nanotubes*
Data source location	*Washington, DC, USA*
Data accessibility	*The data are available with this article*

**Value of the data**

• This data provides a benchmark for carbon nanotubes synthesized via molten electrolyte via the carbon dioxide to carbon nanotube (C2CNT) process useful for comparison to other data on longer length C2CNT wools; specifically:
• C2CNT electrosynthesis with bare (uncoated) cathodes and without pre-electrolysis low current activation.
• C2CNT intermediate length CNTs with intermediate integrated electrolysis charge transfer.
• C2CNT admixing of sulfur, nitrogen and phosphorous (in addition to boron) to carbon nanotubes.
• Scalability of the C2CNT process.

## 1 Data

This data associated with a research study [1] provides a benchmark for carbon nanotubes synthesized using a molten carbonate electrolyte from carbon dioxide using the C2CNT (CO_2_ to carbon nanotube) process useful for comparison to other data on longer length C2CNT wools. The data presented is SEM, TEM and Raman Spectra and economic calculations which provides a benchmark for carbon nanotubes synthesized via molten electrolyte via the carbon dioxide to carbon nanotube (C2CNT) process useful for comparison to other data on longer length C2CNT wools; specifically: (I) C2CNT electrosynthesis with bare (uncoated) cathodes and without pre-electrolysis low current activation. (II) C2CNT Intermediate length CNTs with intermediate integrated electrolysis charge transfer. (III) C2CNT Admixing of sulfur, nitrogen and phosphorous (in addition to boron) to carbon nanotubes, and (IV) Scalability of the C2CNT process.

## 2 Experimental design, materials and methods

### 2.1 C2CNT electrosynthesis with bare (uncoated) cathodes and without pre-electrolysis low current activation

In the initial electrosynthetic methodology ([2], [3] and references therein), the cathode consists of a galvanized (zinc coated) steel electrode, and an initial low current (0.05 A (amps) for 10 min, 0.1 A for 10 min, 0.2 A for 5 min, and 0.4 A for 5 min) series of steps is applied to grow Ni nucleation sites on the cathode, followed by a longer, constant current (controlled at 0.1–0.2 A cm^−2^).

**Fig. 1:**
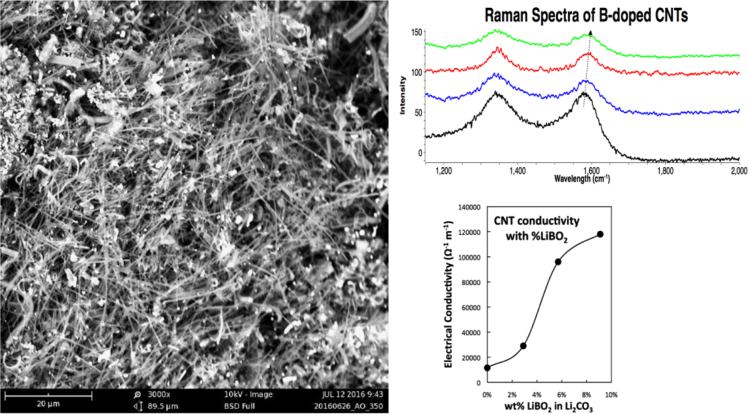
Properties of boron doped CNTs formed by electrosynthesis in molten carbonate. SEM (top) and Raman Spectra (left) of B-doped CNTs formed by 1 Ah electrolysis at a 5 cm^2^ cathode in 5 g LiBO_2_ and 50 g Li_2_CO_3_ at 770 °C. Right: the electrical conductivity of CNTs grown with an increasing concentration of LiBO_2_ dissolved in the Li_2_CO_3_ electrolyte. Note that we had previously reported the anode and cathode surface area each as 10 cm^2^[2], [3]. More specifically this was the total (two sided) exposed surface area, whereas the surface area facing each electrode is 5 cm^2^.

As one example of the initial methodology the electrolysis is conducted with a lithium metaborate additive to the electrolyte, that is to the 50 g of Li_2_CO_3_, either 1.5 g, 3 g, 5 g, 8 g, or 10 g of LiBO_2_ is added to the electrolyte. The SEM observed morphology of the products remains unchanged with these various levels of LiBO_2_ addition, and consists of 5–50 µm long carbon nanotubes (CNTs) as exemplified on the left of Fig. 1. The boron addition to the electrolyte boron-dopes the CNTs (as determined by a Raman peak shift in the G band seen in the top right of Fig. 1) and increases their electrical conductivity by a factor of ten as summarized in the bottom right of Fig. 1.

Several combined carbon dioxide to carbon nanotube (C2CNT) effects (elimination of the cathode zinc coating and pre-electrolysis activation steps, cathode substrate composition, electrolyte aging, and choice of anode substrate composition) lead to the first production of CNT wool. In this data, the first several of these effects (removal of the cathode zinc coating effect, elimination of the pre-electrolysis low-current activation steps and cathode substrate composition choice) are shown. As a first step towards the new synthesis electrolyses are conducted without zinc coated cathodes, which had been considered as a necessary component to the synthesis [2], [3], [4], [5], [6], [7], but instead with fine (3–5 µm) Ni metal powder added directly as additional transition metal to the electrolyte to compensate for the lack of the zinc activating agent. Less than 1% by mass added Ni was sufficient (0.1–10% Ni addition was explored, and larger than 5 µm added Ni powder was less effective than the 2–5 µm powder) to promote CNT growth. Removal of the zinc coating constraint opens the pathway to explore other (than steel) metals as cathode substrates and in particular certain metal substrates promoted a longer CNT product. The previous galvanized steel cathode had a zinc coating and as zinc has a 420 °C melting point which is less than the temperature of the molten electrolyte (≥750 °C) in which the cathode is immersed, liquid zinc could form. In that case the liquid zinc could leave the electrode and helps initiate the dissolution of nickel from the anode or formation of carbon [2], [3], [4], [5]. In lieu of the zinc, the direct, addition of Ni powder to the electrolyte provides sharper control of the initiation of CNT growth than the previous methodology which utilized the release of Ni during the initial gradual formation of a stable Ni oxide layer at the anode which had been observed to require a gradual increase of electrolysis current to yield a high formation of the CNT product at the cathode. Here the higher steady-state electrolysis current can be initially and continuously applied without the need for that lower current density activation of CNT growth.

**Fig. 2:**
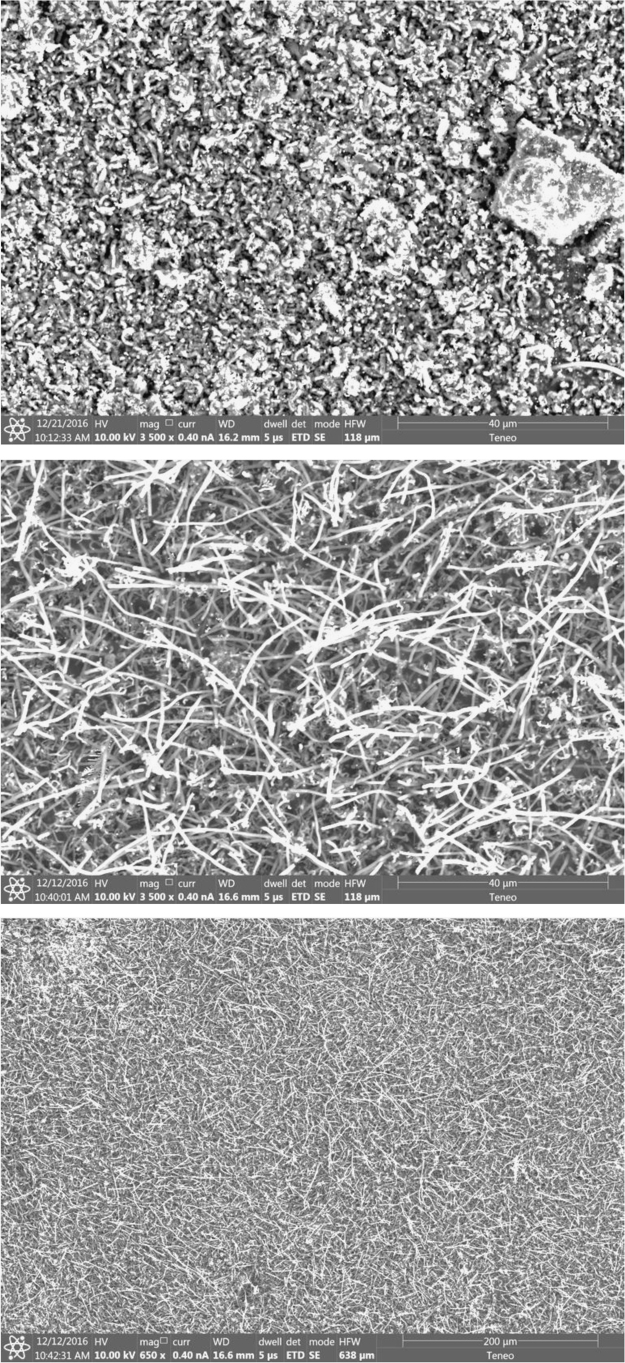
Top: In the absence of added Ni powder, a bare (zinc free) cathode does not form an observable CNT electrolysis product from a fresh molten Li_2_CO_3_ electrolyte, but with appropriate choice of substrate can form a highly uniform CNT product (middle and lower panel) with the addition of a low level (0.1 wt%) 3–5 µm Ni powder to the electrolyte. 1.2 Ah cm^−2^ electrolyses are conducted using an Ir anode (rather than Ni anode, to ensure the anode does not introduce nickel to the electrolyte) and a Monel cathode in 770 °C Li_2_CO_3_.

As seen in Fig. 2 by SEM of the cathode product, even a low level (0.1 wt%) of the Ni powder added to the Li_2_CO_3_ electrolyte promotes CNT growth. To ensure that no Ni is in the system other than that added as Ni powder, an iridium anode, rather than Ni or Ni alloy anode, was used in this electrosynthesis, and we’ve previously noted that Ir is also an effective (albeit expensive) oxygen electrode for the carbonate system. As seen in the top panel of the figure, without the Ni powder (and without the zinc cathode coating) no CNT product is observed. However, with the added Ni powder (and still without the zinc cathode coating), in the middle and lower panels, it seen these product CNTs are highly uniform and of high purity CNTs.

**Fig. 3:**
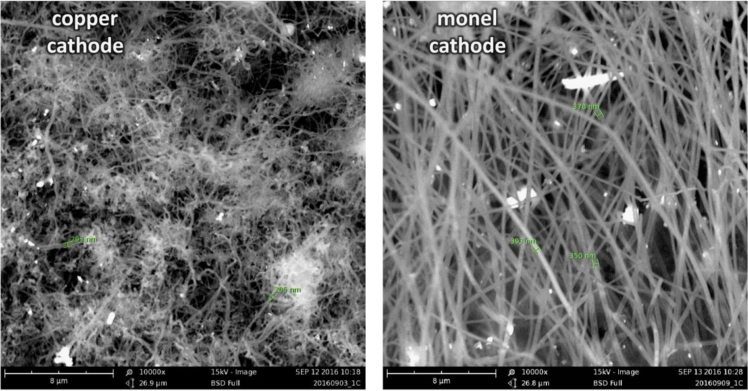
Comparison of the CNT product formed respectively at a copper (left side) and Monel (right side) cathode during short duration 0.3 Ah cm^−2^ electrolysis in 770 °C Li_2_CO_3_.

**Fig. 4:**
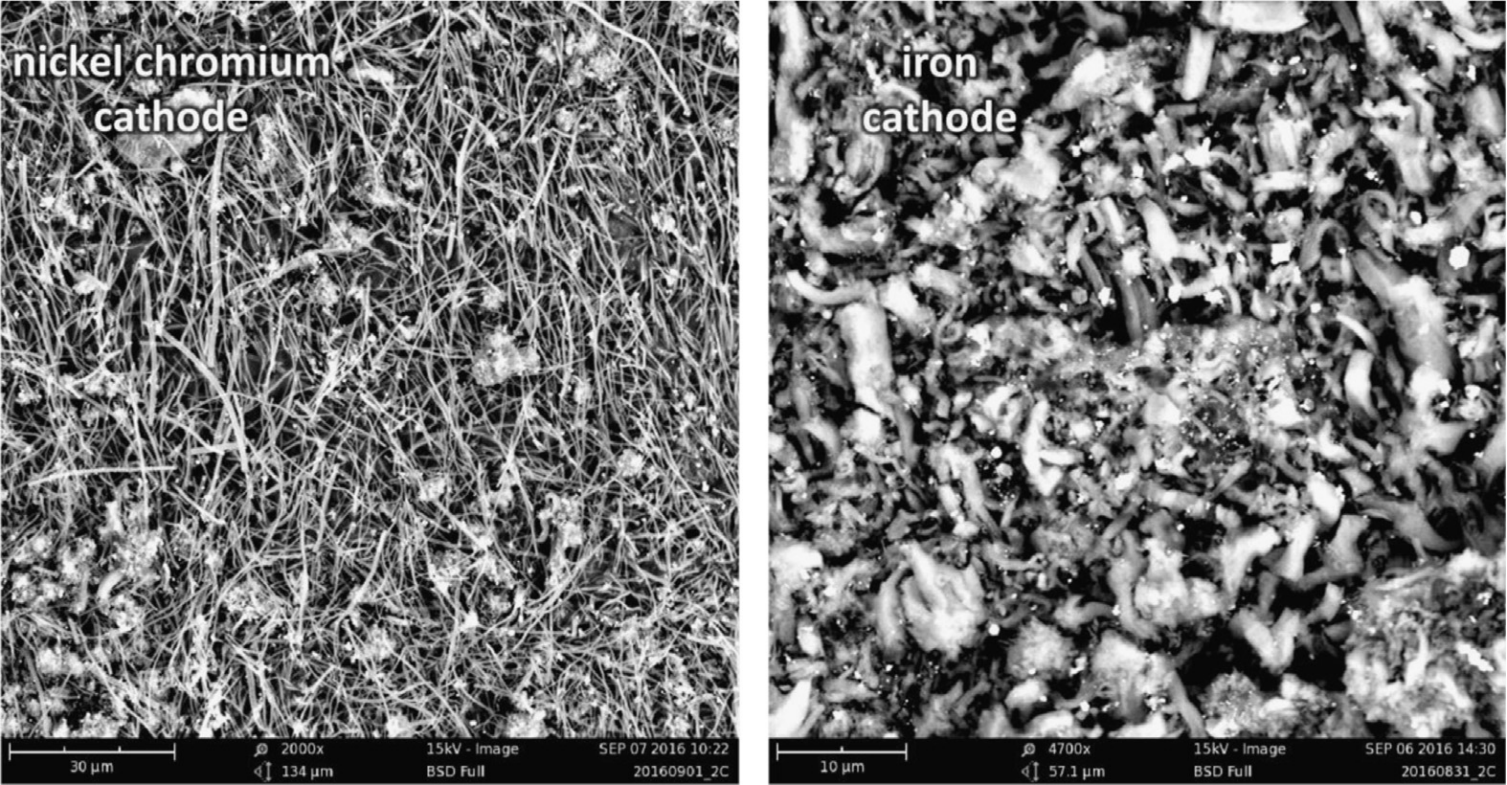
Comparison of the CNT product formed respectively at a nickel chromium alloy (left side) and iron (right side) cathode electrolyses in 770 °C Li_2_CO_3_. The product on Nichrome is formed during intermediate duration (0.8 Ah cm^−2^), while at an iron cathode, as shown even for short duration electrolysis (0.2 Ah cm^−2^), the carbon product is highly heterogeneous when formed.

**Fig. 5:**
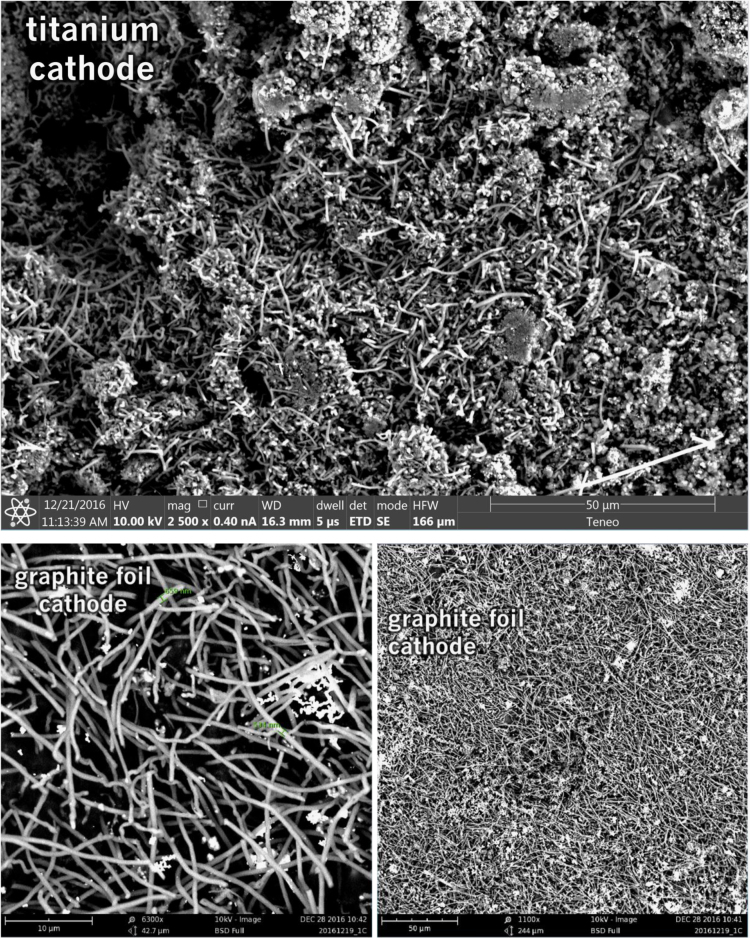
Comparison of the CNT product formed respectively at a titanium (left side) and graphite foil (right side) cathode extended (2.4 Ah cm^−2^) electrolyses in 770 °C Li_2_CO_3_. The graphite foil is cut as a 5 cm^2^ disc, while the titanium (and copper, Monel, iron, steel, nickel or nickel chromium cathode substrate) is coiled wire 5 cm^2^ discs.

Each of the subsequent experiments in this data are conducted on various cathodes, each by electrolysis with 0.4 wt% of 3–5 µm Ni powder utilized in the 770 °C molten lithium carbonate and in each case the electrolyses were conducted at constant current (without an initiating series of activation increasing constant current steps). As seen in Fig. 3, while Monel and copper both produce a high yield of CNTs, the CNT morphology is entirely different after 1.5 h of electrolysis time (at 1-amp constant current between the 5 cm^2^ electrodes). The copper cathode forms thin, tangled CNTs, while the Monel cathode forms uniform, thicker and straight CNTs. A pure Ni cathode (not shown) produces a CNT similar to that of copper although the CNT yield (80–85%) is less than that of the ≥85% yield of the copper cathode. As shown on the left side of Fig. 4, a Nichrome cathode provides straighter CNTs than a pure nickel cathode. The nickel chromium cathode continues to produce straight CNTs during intermediate duration electrolyses, but unlike the Monel cathode CNTs from a nickel chromium substrate cathode did not continue to grow during extended electrolytes. Iron oxide (unlike nickel oxide) is highly soluble in molten carbonate and we have previously shown that its addition to the electrolyte generates an uncontrolled growth of a profusion of nanostructures [2], [3], [6]. A pure iron cathode substrate generates a similar heterogeneous product as seen in the right side of Fig. 4. Fig. 5 presents the product generated at several different cathodes subsequent to extended (12 Ah) electrolyses. As seen a titanium cathode yielded shorter and only moderate quality CNTs, while a graphite foil cathode yielded high quality, but shorter (than a Monel Cathode) CNTs subsequent to these extended electrolysis.

Of the eight cathodes examined (Monel, steel, iron, nickel, nickel chromium, copper, titanium, and graphite) only Monel exhibited a CNT product whose length increases (and was approximately linear) with the increase in integrated electrolysis charge density. Another eight other similar alloys from Online Metals (online metals.com) were acquired for further data comparison consisting of:

(i) Nickel silver (55% Cu, 27% Zn, 18% Ni) from Online Metals (online metals.com)
(ii) Brass 260 (70% Cu, 30% Zn) Online Metals
(iii) Naval Brass 464 (61% Cu, 39.25% Zn, 0.75% Sn) Online Metals
(iv) Ni K500 (>63% Ni, 27–33% Cu, 2.3–3.15% Al, max 2.0% Fe, 1.5% Mn, 0.5% Si) Online Metals
(v) Cu 182 (99.1% Cu, 0.9% Cr) Online Metals
(vi) Munz metal (brass) (61% Cu, 40% Zn, trace iron) Online Metals
(vii) Cu 715 (30% Ni, 70% Cu) MARMETAL
(viii) Cu 706 (10% Ni, 90% Cu) MARMETAL

Subsequent to 770 °C molten lithium carbonate electrolysis the Munz brass produces CNTs of macroscopic dimensions approaching, but not as large as, the Monel cathode, as another example the 30% Ni, 70% Cu alloy produces a single product CNTs containing both short and macroscopic reminiscent of both pure Cu and Monel.

Subsequent to the initial screening of the electrolysis cathode substrate, two further significant advances to the C2CNT process were found. 1) Uncoated (bare, without zinc) substrate cathodes can generate a CNT product without any Ni powder added to the electrolyte, when the molten electrolyte is “aged” (left molten prior to electrolysis) for 24 h prior to electrolysis (longer periods did not further improve CNT quality). The low level of nickel dissolving from the anode is sufficient to migrate and act as nucleation at the cathode, and this originates while the anodic nickel oxide layer is established at the start of the electrolysis. 2) The quality (length and quantity of CNT) improves when the anode consists of Nichrome (Nichrome 60, also referred to as Chromel C (60% Ni, 16% Cr, 24% Fe)), rather than a pure nickel anode, and both Ni and Cr are then observed by EDS at the cathode CNT nucleation sites.

The physical chemical environment of the conventional CVD CNT synthesis is different than that of the new C2CNT synthesis in most aspects. The latter is an electrochemical process, while the former is chemical. The latter utilizes CO_2_, while the former utilizes organics as the reactant, and the latter occurs at the liquid/solid interface, while the latter generally occurs at a gas/solid interface. There are also significant subtle differences. C2CNT provides a higher density of reactive carbon (the molten carbonate electrolyte) near the growth interface, and while an electric field may, or may not, be applied to the substrate during CVD CNT growth, there is always an intense electric field rapidly decreasing through the double layer adjacent to the cathode during C2CNT growth. Despite these differences, it is phenomena that several phenomena here that promote C2CNT processes also appear to have a similar effect on CVD CNT growth processes, and other CVD advances suggest pathways for further C2CNT improvements. Few-layered graphene/multiwalled CNT structures have been observed to form by CVD metal (Ni) substrates [8]. In CVD, copper containing substrates can improve carbon mobility that improves the uniformity of the base graphene layer to initiate CNT growth [9]. In CVD, larger multiwall CNT (more than ten walled MWCNTs) almost exhibit tip growth with the nucleating metal in the lead, rather than base growth as it is thought be that larger nano-particles adhere less to substrate, while the growth of few walled MWCNTs (≤7 walls) are dominated by base growth [10]. We suggest this is particularly applicable to the new electrochemical C2CNT process as the tip growth also maximizes exposure to the bulk electrolyte and minimizes bulk carbon(IV) diffusion to the growing CNT. In CVD, rapid pre-heating of the catalyst before introduction of the feed gas resulted in the growth of longer/higher yield CNTs, and applied electric field helped promote the growth of more aligned and straighter CNTs [11]. In CVD, metal nano-particles can grow and become trapped or move along within the CNT, consuming and requiring more catalyst or stopping the growth process completely [12]. In single walled CNT CVD growth studies, a nucleation period of 5–10 s occurs at the start of the CNT growth. Initially, this nucleation period requires more carbon in proximity to the growth region and less during the next period consisting of a simultaneous CNT growth and repair stage [13]. The growth of longer CNTs was facilitated by the initial preparation of micrometer catalyst islands on a substrate CNT [14]. Certain pairs of bi-metallic catalysts promote CNT growth 10–100 fold better than single metals alone, and splitting the catalyst into two groups, ones that help nucleation more and one that helps growth and repair more leads to the best metal pairs (choose the best from each group), yielding the organization [15]:

1. (Order of) best: Ni+Co>Ni+Pt≫Cu+Co
  Ni+Co>Ni+Fe~Ni≫Fe
  Ni+Co>Ni+Pt>Ni+Cu
2. Nucleation: Co>Pt>Ni slightly less>Cu (poisons)
  Theory: Mo>Cr>Co>Pt>Re>Fe>Ni>Pd
3. Growth and repair: Ni>Co>Pt> Cu
  Theory: Ni+Mo>Ni+Cr>Ni+Co>Ni+ Pt>Ni+Rd>Ni +Fe>Ni>Fe
  and Fe+Mo>Fe+Cr>Fe+Co>Fe+Pt>Fe +Rh>Fe
  and Ni+Mo>Fe+Mo>Co+Mo>Co (2-4 by theory, later Co+Mo>Co

Different metals lead to different diameter CNTs [16]. Islands were found to form on the substrate and their size can lead to optimization [17]. The main article [1] interprets this observed data parallel between prior CVD CNT syntheses and our new molten carbonate C2CNT synthesis and provides a growth mechanism for the latter process.

### 2.2 C2CNT intermediate length CNTs with intermediate integrated electrolysis charge transfer

**Fig. 6:**
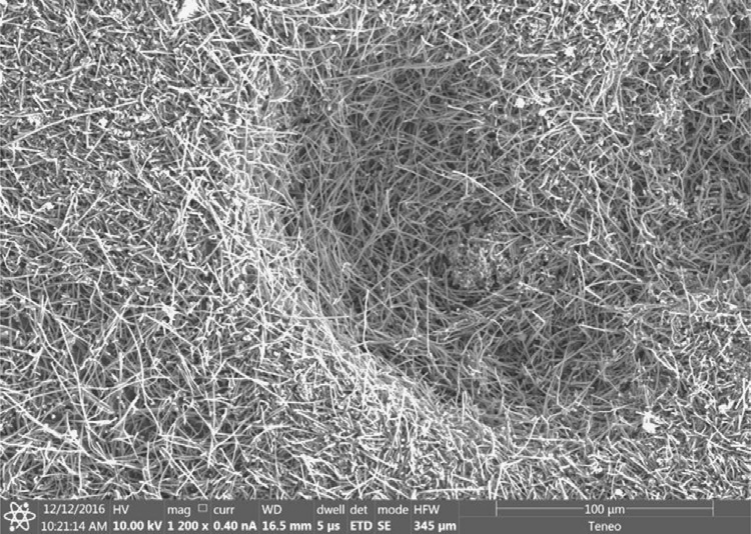
SEM of B-doped CNTs formed by 6 Ah electrolysis at a 5 cm^2^ Monel cathode in 5 g LiBO_2_ and 50 g Li_2_CO_3_ at 770 °C.

Fig. 6 presents an intermediate stage of the C2CNT synthesis advancement resulting in improved CNT yield and improved CNT length. In this intermediate advancement of synthesis, a bare Monel (rather than galvanized steel) substrate was used, a 2-step (0.05 A/13 min, 0.25 A/12 min), rather than the original 4-step electrolysis pre-activation was utilized, and 0.4 g (0.8 wt%) of Ni powder was added to the 50 g of 770 °C Li_2_CO_3_. electrolyte, and an Ir anode, and a somewhat higher current density and integrated charge was used was used (1 A through the 5 cm^2^ electrode (0.2 A cm^−2^), for 6 h (integrated charge of 1.2 Ah cm^−2^). The higher constant current resulted in a higher (1.6 V) potential during the electrolysis. Up to 10 wt% added LiBO_2_ was observed to have no effect on the observed CNT morphology, but as described in Section 2.1 enhances their electrical conductivity. The electrolysis product shown in Fig. 6 contains 3 g LiBO_2_ added to the electrolyte. As seen in the figure, even without the additional improvements of mixed Ni/Cr, rather than just Ni, nucleation, and without electrolyte aging, the CNT quality is high and the tangled CNTs are longer, ranging from 20 to over 200 µm in length.

### 2.3 C2CNT admixing of sulfur, nitrogen and phosphorous (in addition to boron, Section 2.1) to carbon nanotubes

**Fig. 7:**
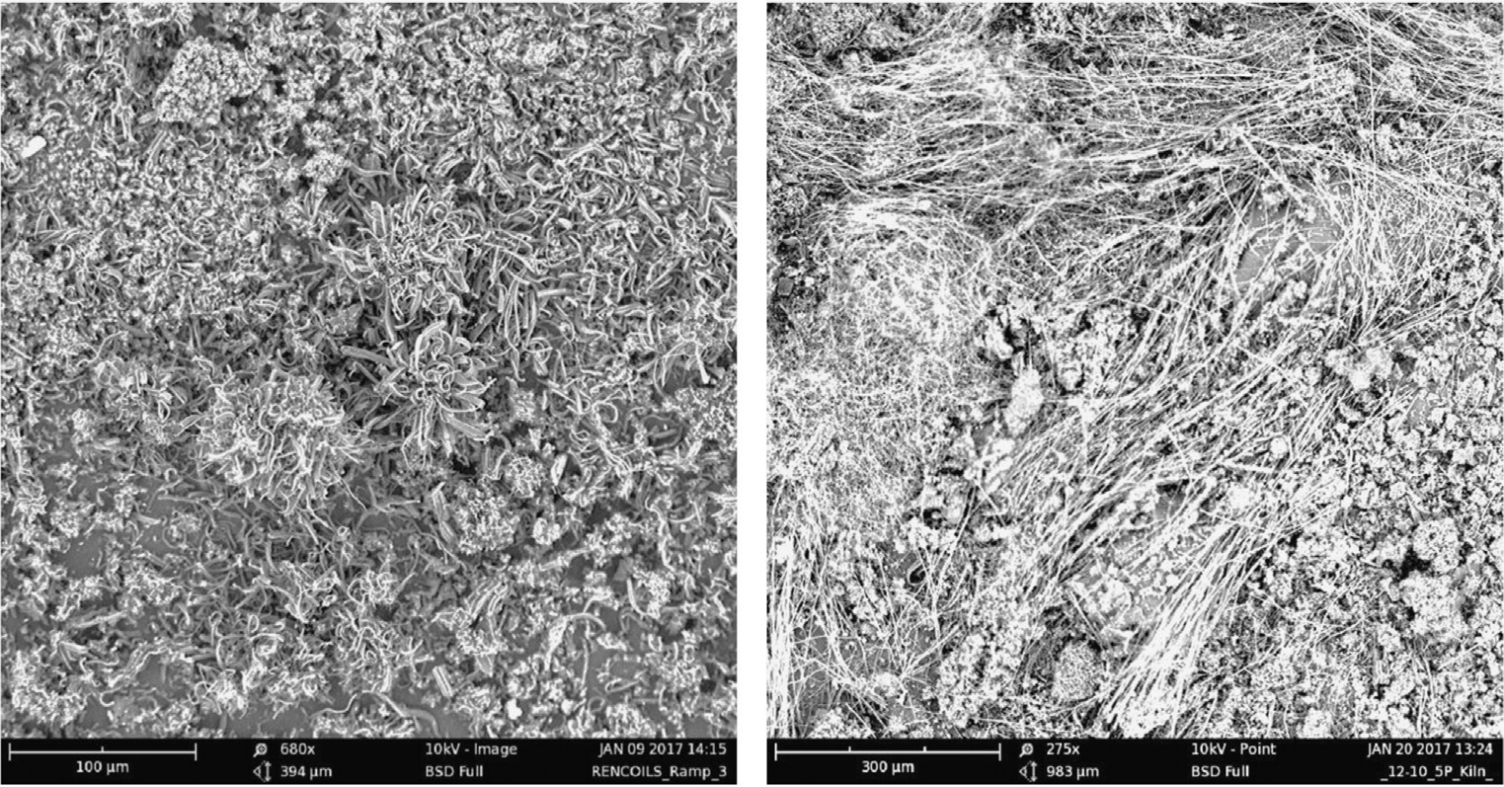
Left: SEM of the S-heteroatom product formed by 0.4 Ah cm^−2^ electrolyis at a galvanized steel cathode in 50 g of 770 °C Li_2_CO_3_ containing 0.074 g Li_2_SO_4_. Right: SEM of the P-heteroatom product formed by 0.8 Ah cm^−2^ electrolyis at a Monel cathode in 50 g of 770 °C Li_2_CO_3_ containing 0.5 mol% Li_2_PO_4_.

Here, we show the first single step electrosynthesis of sulfur heteroatom CNTs. Sulfur doped carbons, including CVD grown CNTs, have a variety of applications [18], [19]. Unlike in pure carbonate [3], previously no carbon product (CNT or otherwise) was observed to form at the cathode during the electrolysis of 1 mol% (or 3, or 5 mol%) Li_2_SO_4_ in 770 °C Li_2_CO_3_. The observed potentials at 1 A are lower with higher [Li_2_SO_4_] (and are lower than the 1–2 V electrolysis potential observed without Li_2_SO_4_). This lack of CNT formation is in accord with the electronegativity of sulfur compared to carbon, which favors the thermodynamic formation of the former compared to the latter. To improve the energetics of carbon formation, the concentration of sulfate is decreased (relative to carbonate) creating a pathway to the observed formation of sulfur containing CNTs. Specifically, the Fig. 7's left side presents sulfur containing CNTs from molten carbonate electrolysis with 0.1 mol% sulfate subsequent to a 2-h electrolysis at 1 A (using the conventional galvanized steel cathode and Ni 200 wire anode and without added Ni metal powder). EDS of the CNT product measured 0.1 mol% of sulfur in the CNT product. As in previous experiments, prior to this higher current extended electrolysis, cathode nucleation was facilitated by an application of lower constant currents sequentially applied (each for 10 min) and increased from 0.05, 0.10, 0.25 to 0.5 A. As previously observed with successful (non-sulfur containing electrolyte) CNT electrolyses [2], [3], [4], [6], [7], the initial 10-min lowest current electrolysis occurred at a potential of 0.4–0.5 V, which is consistent with the expected nucleation by Ni on the cathode while each of the subsequent increasing constant currents occurred at increasing potentials between 1 and 2 V.

The previous electrolytic formation of P-heteroatom CNTs from lithium metaphosphate dissolved in a lithium carbonate electrolyte [3] is improved here, including the first evidence of phosphorous in the CNT product. The use of LiPO_3_ is observed to facilitate salt dissolution in the lithium carbonate electrolyte. Variations which led to the improved length and yield of P-containing CNTs include an increase from the previous 1% to 5 mol% LiPO_3_**,** and the use of a Monel, rather than galvanized steel cathode. On the right side of Fig. 7, the product P-heteratom long (300–600 µm) are produced with intermediate 0.8 Ah cm^−2^ charge at a low current density of 0.03 A cm^−2^; a conventional (Ni 200) anode and no Ni powder was added to the electrolyte during this C2CNT synthesis, EDS of the CNT product measured 0.3 mol% of phosphorous in the CNT product. This is substantially lower than the electrolytic concentration of phosphorous, and the P-heteroatom may provide a poor lattice match to the CNT.

As with boron, sulfur and nitrogen doping, nitrogen doping of CNTs can lead to a variety of specialized applications (Ren et al., 2017). An N-CNT product is also observed from electrolysis of LiNO_3_ in the 770 °C Li_2_CO_3_ electrolyte. In this case, the yield of CNTs improves with a 5 mol%, compared to a 1 mol%, dissolution of LiNO_3_ within the electrolyte. Presumably, the added, dissolved lithium nitrate equilibrates to lithium nitrite in the molten electrolyte, analogous to the known solid state thermal decomposition:

(1) Solid LiNO_3_ thermal decomposition occurs above 500°C: LiNO_3_→LiNO_2_+1/2O_2_

Subsequent to electrolysis, EDS analysis of nitrogen within the product is indicated, but not confirmed, as the EDS instrumentation was broad and not capable or resolving the near lying carbon (12.01) and nitrogen (14.01) peaks. SEM and an extended analysis of the nitrogen CNT product, and also a detailed analysis of the doped CNT growth mechanism, further elemental probes of each of the heteroatom modified CNTs, and applications of these CNTs will each be presented in an expanded, future investigation.

The electrosynthesis of CNTs containing the heteroatoms of sulfur, phosphorus or boron, and likely nitrogen, is observed. Doped CNTs can have unusual, useful properties including high electrical conductivity, catalysis, heavy metal removal, enhanced oxygen kinetics and improved charge storage.

### 2.4 Scalability and cost of the C2CNT process

**Fig. 8:**
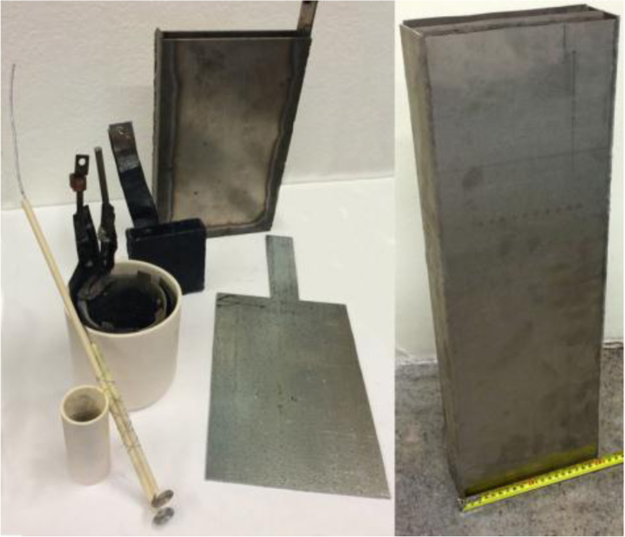
The evolution of the electrolysis chamber. Earlier versions can be seen in the front on the left, and later versions in the back and to the right. The rectangular electrolysis chambers use the interior walls as the air electrode.

As an electrochemical process, C2CNT is linearly scale-able with our increasing area of the electrolysis chamber, Fig. 8.

**Fig. 9:**
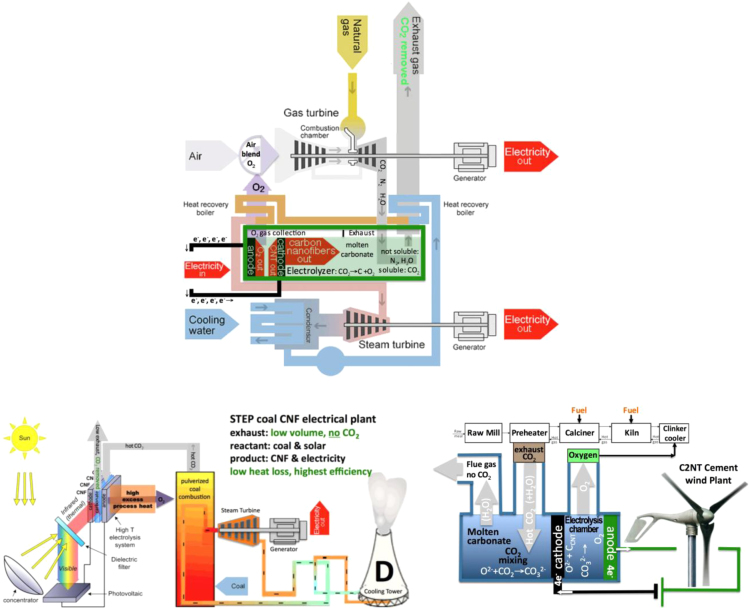
Top: Schematic of a CNT combined cycle power plant Lau et al. [20]. Middle: Transforming CO_2_ emissions from a coal combustion plant into CNTs using solar energy Lau et al. [20]. Bottom: C2CNT Cement wind plant: The full oxy-fuel configuration is shown. The plant does not emit CO_2_, and over time cement produced absorbs CO_2_. Hence the process is carbon negative, which compares favorably to the large positive carbon signature of conventional cement plants [21].

The thermodynamic and cost savings of new and retrofit gas, cement, and coal power plants has been analyzed [20], [21]. Industrial plant retrofit provides a ready source of hot CO_2_ for electrolysis, and the added the oxy-fuel energy benefits (in addition to the benefit of CO_2_ removal and the production of CNTs) of the C2CNT co-product O_2_ looped back into the plant, the value of the CNT product is illustrated in Fig. 9. The top of the figure shows this oxy-fuel advantage with a gas combined cycle power plant. The bottom right and left of the figures show this oxy-fuel advantage respectively, in cement or coal power or cement plants. In the lower portion of the figure, the electrical power for the electrolysis C2CNT component is provide by renewable energy (solar or wind power, respectively), as opposed to utilizing electricity from the fossil fuel (gas) power plant. It should be noted that conventional deleterious coal plant sulfur and nitrogen emissions might instead be beneficial, when considered as a source of CNT heteroatom doping as described in Section 2.3. Such gas, coal and cement plants provide further impetus for substantial intermediate C2CNT scale-up en route to direct atmospheric transformation of CO_2_–CNTs.

Cost analysis here is structured on eliminating (by transformation to CNTs) the CO_2_ exhaust from a gas, coal or cement plant [20], [21]. The cost incorporates the increased fuel combustion efficiency in these plants when the C2CNT oxygen product (CO_2_→CNT+O_2_) is looped back into the plant's fuel combustion [22].

This analysis is derived from a comparison to the known cost structure of a comparable, mature industry: aluminum production. The C2CNT process bears many similarities to aluminum smelting. Both processes consist of molten electrolysis, and do not use noble or exotic materials. Aluminum smelting produces aluminum metal from alumina (using bauxite, sodium hydroxide and electricity), while C2CNT produces carbon nanotubes from carbon dioxide (using carbon dioxide and electricity). Aluminum smelting operates at 960 °C in a molten cryolite electrolyte. The C2CNT process operates under somewhat milder conditions at 770 °C in a less exotic, molten carbonate electrolyte. Both processes operate at high rate (hundreds of mA per cm^2^) and low polarization. In both cases the electrolysis chamber consists of common metals, common insulators (such as kiln or “firebricks”), and control equipment. Electrolysis in the Aluminum smelting process is driven at approximately 4 V using 3 electrons per aluminum. The C2CNT electrolysis is driven at approximately 1 V using 4 electrons per carbon dioxide. C2CNT and aluminum smelters have approximately equivalent output (tonnage) rates.

Aluminum costs ~$1880 per metric ton, of which ~32% of the cost is electricity [23]. Today's newer, more efficient Al plants require 12 MW h per ton, whereas older Al plant require 15 MW h per ton. 13 MW h is measured and calculated from a 94% efficient 3 electron per Al electrolysis at 4.1 V [24]. $600 for 12 MW h=$0.05 per kW h. This Al electricity cost varies from lowest (in the Middle East) at $300, to mid range $650 (US), to highest (China with high energy tariffs) of $1020/ton.

**Fig. 10:**
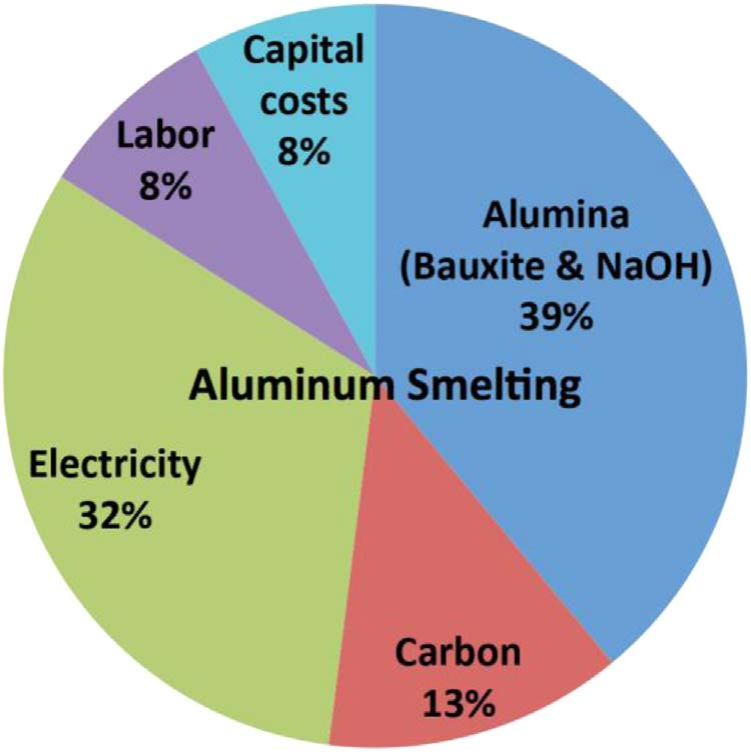
Aluminum smelter cost structure (modified from [23]).

A breakdown of Al production costs per metric ton (tonne) of Al, based on market costs are summarized in Fig. 10 and Table 1, and consists of: Consumable Expenses (32% electricity and 52%; reactants=84%), Electricity: 32%, Labor: 8%, and Capital Expenses (amortized cost of electrolyzers, processing equipment, and miscellaneous overhead). Note that the energy to drive the aluminum production originates from two sources (electricity and energy released from the consumed carbon anode).

**Table 1:** Comparison of aluminum and C2CNT production costs.

Process	$US Cost (% of total)					
	Alumina	Carbon	Electricity	Labor	Capital	Total
Aluminum	733(39%)	$244(13%)	$602(32%)	$150 (8%)	$150 (8%)	$1880(100%)
	CO_2_	–	Electricity	Labor	Capital	Total
C2CNT	$0	$0	$360	$150	$150	$660

As compared in Table 1, and unlike Al smelting, the C2CNT process uses a no-cost oxide as the reactant (carbon dioxide, rather than bauxite). Both are straightforward, high current density electrochemical (molten electrolytic reduction of oxide) processes. The C2CNT process operates under somewhat milder conditions at 770 °C in a less exotic, molten carbonate electrolyte at similar rates of output, and to a first order of approximation, both processes will be assumed to have the same labor and capital costs. Whereas, Al production requires ~13 MW h per ton of aluminum, C2CNT production requires less energy (7 MW h) per ton of carbon nanotubes. This energy is calculated here from the C2CNT 1 V electrolysis consuming 4 electron per carbon dioxide splitting efficiency. The observed electrolysis voltage varies from 0.8 V to up to 2 V, decreasing with higher concentrations of added lithium oxide, and increasing with current density and with mixed molten carbonate electrolytes [2], [28]. Using the formula weight to convert mass carbon dioxide to moles, and Faradays constant at 1 V yields 2.4 MW h per ton CO_2_, which decreases to 2.0 MW h per ton CO2 (which is 7.2 MW h per CNT) by the 20% energy recovered through driving the turbine more efficiently with pure oxygen (looped in from the C2CNT electrolysis), rather than regular air, combustion (Lau, 2016). This yields an electrical cost of $360 per ton CNT, and as summarized in Table 1, a total cost of $660 per ton of CNT. The electrical cost falls per ton CNT based on less expensive wind electric, equivalent to (x12.01/44.01) $50 per ton of CO_2_ (Licht, 2017). Higher production rates will increase this cost, while the imposition of a carbon tax or carbon credits will lower this cost.

**Fig. 11:**
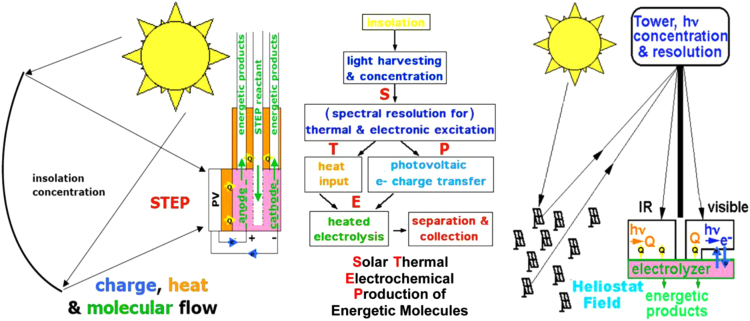
Global use of sunlight to drive the formation of energy rich molecules. Left: Charge, and heat flow in STEP: heat flow (yellow arrows), electron flow (blue), and reagent flow (green). Right: Beam splitters redirect sub-bandgap sunlight away from the PV onto the electrolyzer Licht et al. [26].

C2CNT atmospheric mitigation does not require pre-concentration of the CO_2_. Heat is then provided using photovoltaic discarded thermal sunlight [25], [26], [27] as illustrated in Fig. 11.

The following section is expanded by addition of a CO_2_ availability concluding paragraph from “Scalability of STEP Processes” section of [26], which in turn was expanded from the text and Supporting Information from [25]. Note, since this 2011 analysis, subsequent total estimates of the extent of CO_2_ released in industrial revolution have increased to over 1.1 teratons.

STEP can be used to remove and convert CO_2_. As with water splitting, the electrolysis potential required for CO_2_ to CO splitting falls rapidly with increasing temperature, and we have shown that a photovoltaic, converting solar to electronic energy at 37% efficiency and 2.7 V, may be used to drive three CO_2_ splitting, lithium carbonate electrolysis cells, each operating at 0.9 V, and each generating a 2 electron CO product. The energy of the CO product is 1.3 V (Eq. (1)), even though generated by electrolysis at only 0.9 V due to synergistic use of solar thermal energy. At lower temperature (770 °C, rather than 950 °C), carbon, rather than CO, is the preferred product, and this 4 electron reduction approaches 100% Faradaic efficiency.

The CO_2_ STEP process consists of solar driven and solar thermal assisted CO_2_ electrolysis. Industrial environments provide opportunities to further enhance efficiencies; for example fossil-fueled burner exhaust provides a source of relatively concentrated, hot CO_2_. The product carbon may be stored or used. STEP represents a new solar energy conversion processes to produce energetic molecules. Individual components used in the process are rapidly maturing technologies including wind electric, molten carbonate fuel cells, and solar thermal technologies.

It is of interest whether material resources are sufficient to expand the process to substantially impact (decrease) atmospheric levels of CO_2_. The buildup of atmospheric CO_2_ levels from a 280–392 ppm occurring over the industrial revolution comprises an increase of 1.9×10^16^ mol (8.2×10^11^ metric tons) of CO_2_, and will take a comparable effort to remove. It would be preferable if this effort produces useable, rather than sequestered, resources. We calculate below a scaled-up STEP capture process can remove and convert all excess atmospheric CO_2_ to carbon.

In STEP, 6 kW h m^−2^ of sunlight per day, at 500 suns on 1 m^2^ of 38% efficient CPV, will generate 420 kAh at 2.7 V to drive three series connected molten carbonate electrolysis cells to CO, or two series connected series connected molten carbonate electrolysis cells to form solid carbon. This will capture 7.8×10^3^ mol of CO_2_ day^−1^ to form solid carbon (based on 420 kA h ⋅ 2 series cells/4 Faraday mol^−1^ CO_2_). The CO_2_ consumed per day is three fold higher to form the CO product (based on 3 series cells and 2 F mol^−1^ CO_2_) in lieu of solid carbon. The material resources to decrease atmospheric CO_2_ concentrations with STEP carbon capture, appear to be reasonable. From the daily conversion rate of 7.8×10^3^ mol of CO_2_ per square meter of CPV, the capture process, scaled to 700 km^2^ of CPV operating for 10 years can remove and convert all the increase of 1.9×10^16^ mol of atmospheric CO_2_ to solid carbon. A larger current density at the electrolysis electrodes, will increase the required voltage and would increase the required area of CPVs. While the STEP product (chemicals, rather than electricity) is different than contemporary concentrated solar power (CSP) systems, components including a tracker for effective solar concentration are similar (although an electrochemical reactor, replaces the mechanical turbine). A variety of CSP installations, which include molten salt heat storage, are being commercialized, and costs are decreasing. STEP provides higher solar energy conversion efficiencies than CSP, and secondary losses can be lower (for example, there are no grid-related transmission losses). Contemporary concentrators, such as based on plastic Fresnel or flat mirror technologies, are relatively inexpensive, but may become a growing fraction of cost as concentration increases. A greater degree of solar concentration, for example 2000 suns, rather than 500 suns, will proportionally decrease the quantity of required CPV to 175 km^2^, while the concentrator area will remain the same at 350,000 km^2^, equivalent to 4% of the area of the Sahara Desert (which averages ~6 kW h m^−2^ of sunlight per day), to remove anthropogenic CO_2_ in ten years.

700 km^2^ of CPV plant will generate 5×10^13^ A of electrolysis current, and require ~2 million metric tonnes of lithium carbonate, as calculated from a 2 kg/liter density of lithium carbonate, and assuming that improved, rather than flat, morphology electrodes will operate at 5 A/cm^2^ (1000 km^2^) in a cell of 1 mm thick. Thicker, or lower current density, cells will require proportionally more lithium carbonate. Fifty, rather than ten, years to return the atmosphere to pre-industrial CO_2_ levels will require proportionally less lithium carbonate. These values are viable within the current production of lithium carbonate. Lithium carbonate availability as a global resource has been under recent scrutiny to meet the growing lithium battery market. It has been estimated that the current global annual production of 0.13 million metric tons of LCE (lithium carbonate equivalents) will increase to 0.24 million tons by 2015. Alternative, mixed alkali/alkali earth carbonates are also suitable. Sodium carbonate is substantially more available, and as noted can be combined with lithium carbonate for molten CO_2_ splitting. Low velocity natural wind speeds are sufficient to move this air to C2CNT processors. A 100 km by 100 km area with wind moving through it at 2 km per h will deliver over a teraton of CO_2_ during a decade.
